# Constitutive heterochromatin formation and transcription in mammals

**DOI:** 10.1186/1756-8935-8-3

**Published:** 2015-01-15

**Authors:** Nehmé Saksouk, Elisabeth Simboeck, Jérôme Déjardin

**Affiliations:** INSERM AVENIR Team, Institute of Human Genetics, CNRS UPR 1142, Montpellier, France

**Keywords:** epigenetic factors, heterochromatin, histone modifying enzymes, pericentromere, transcription

## Abstract

Constitutive heterochromatin, mainly formed at the gene-poor regions of pericentromeres, is believed to ensure a condensed and transcriptionally inert chromatin conformation. Pericentromeres consist of repetitive tandem satellite repeats and are crucial chromosomal elements that are responsible for accurate chromosome segregation in mitosis. The repeat sequences are not conserved and can greatly vary between different organisms, suggesting that pericentromeric functions might be controlled epigenetically. In this review, we will discuss how constitutive heterochromatin is formed and maintained at pericentromeres in order to ensure their integrity. We will describe the biogenesis and the function of main epigenetic pathways that are involved and how they are interconnected. Interestingly, recent findings suggest that alternative pathways could substitute for well-established pathways when disrupted, suggesting that constitutive heterochromatin harbors much more plasticity than previously assumed. In addition, despite of the heterochromatic nature of pericentromeres, there is increasing evidence for active and regulated transcription at these loci, in a multitude of organisms and under various biological contexts. Thus, in the second part of this review, we will address this relatively new aspect and discuss putative functions of pericentromeric expression.

## Review

The observation of differential chromosomal staining by Heitz in 1928 forms the basis of the categorization of eukaryotic genomes into two major functional states. Euchromatin corresponds to a rather open and transcriptionally active conformation, while heterochromatin designates a condensed and transcriptionally inert conformation. A major function of heterochromatin is to protect the underlying DNA from being accessed by dedicated machineries and, thus, used for transcription or for other DNA-based transactions, such as repair. Heterochromatin has been further categorized into facultative and constitutive heterochromatin. Facultative heterochromatin refers to a type that may form at various chromosomal regions, which usually contain genes that must be kept silent upon developmental cues. In contrast, constitutive heterochromatin is believed to occur at the same genomic regions in every cell type and these regions usually do not contain genes. Hence, constitutive heterochromatin is often viewed as a more static structure than facultative heterochromatin. In most organisms, the bulk of constitutive heterochromatin forms at pericentromeric regions and at telomeres, and these gene-poor areas are usually made of tandem repetitions, also named satellites, that vary in size from 5 bp to a few hundred bp (reviewed in [1, 2]).

The biochemical and early genetic characterizations of players acting to promote or counteract heterochromatin formation are at the foundation of modern epigenetics. Heterochromatin is characterized by typical post-translational modification profiles on histones. The combination of these marks is ‘read’ and translated into biological outputs by dedicated protein machineries. The most prominent histone feature in heterochromatin is global hypoacetylation, which leads to chromatin fiber compaction. In addition, specific methylation marks are also enriched. A typical mark of constitutive heterochromatin is the trimethylation of histone H3 on lysine 9 (H3K9me3), while H3K27me3 is usually enriched on facultative heterochromatin. As discussed in this review, both marks recruit distinct protein machineries and may underlie distinct biological features, although the consequence is chromatin compaction in both cases.

In most metazoans, telomeres are constituted by a repeated short conserved DNA motif (5′-TTAGGG-3′) and harbor enrichment in H3K9me3. Telomeres are bound by conserved protein machineries acting to protect chromosomal ends from being recognized as double-strand breaks. The conservation of both DNA sequences and bound machineries suggests that major telomeric functions might not critically rely on epigenetic mechanisms. Thus, while undoubtedly playing a role, heterochromatic activities at telomeres will not be detailed in this review.

**Figure 1:**
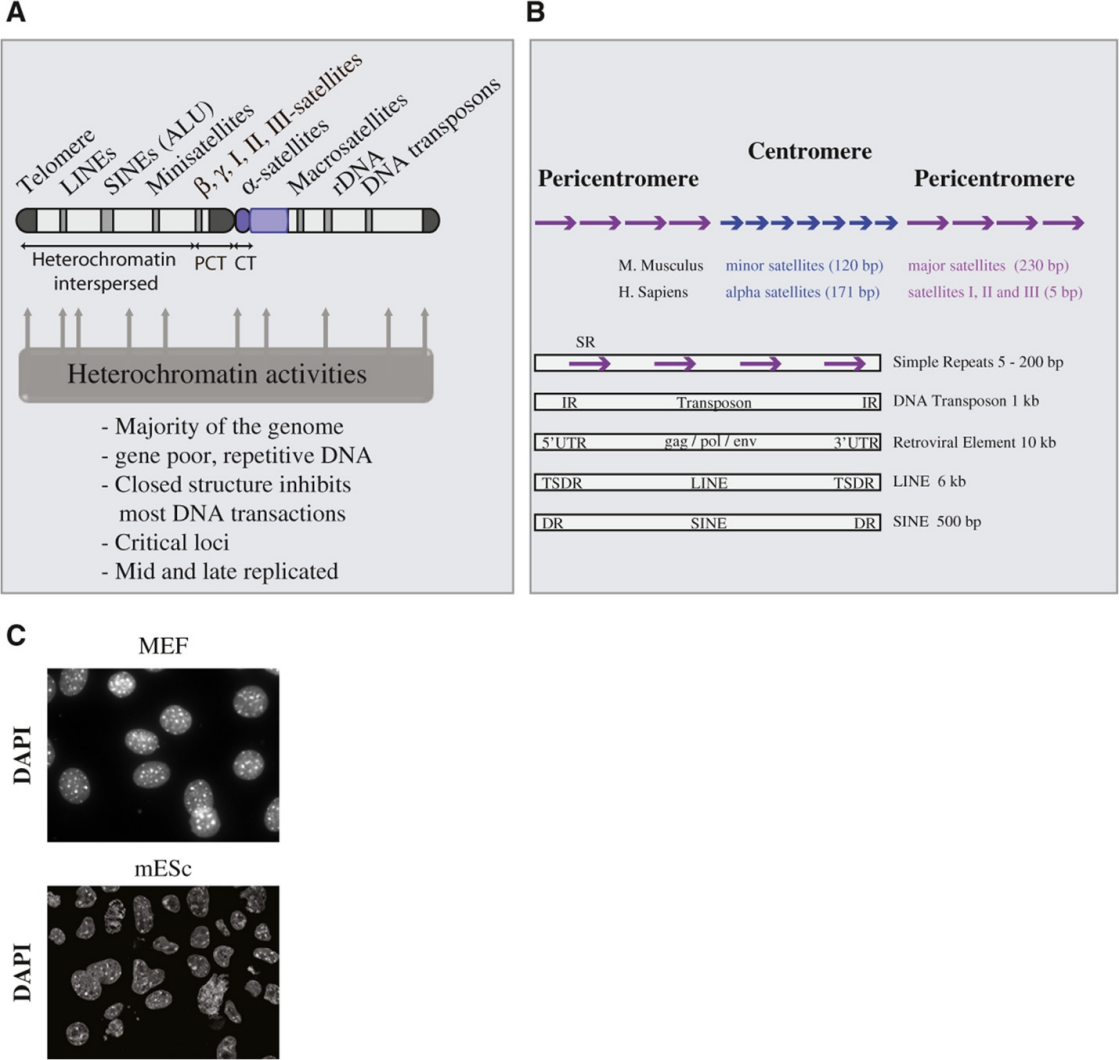
**Organization of constitutive heterochromatin. (A)** Constitutive heterochromatin is found at pericentromeric, telomeric, and ribosomal regions, as well as at different loci along the chromosome **(B)** Centromeres and pericentromeres consist of predominantly repetitive DNA sequences, including simple repeats, DNA transposons, LTR-endogenous retroviral elements, non-LTR autonomous retrotransposons including long interspersed elements and short interspersed elements. The approximate length of the different repetitive elements is indicated. In mice, centromeres consist mainly of minor satellites and pericentromeres of major satellites. In human beings, centromeres consist mainly of alpha satellites and pericentromeres of chromosome specific satellite repeats, including satellites I, II and III. **(C)** Chromocenters in differentiated cells are smaller, more numerous and more condensed than chromocenters in undifferentiated pluripotent cells, which are probably more dynamic. CT, centromere; DR, direct repeat; IR, inverted repeats; LINE, long interspersed element; MEF, mouse embryonic fibroblasts; mESC, mouse embryonic stem cells; PCT, pericentromere; SINE, short interspersed element; TSDR, target site direct repeat.

The bulk of constitutive heterochromatin forms at pericentromeric regions (Figure 1A). In contrast with telomeres, the repeat sequences making pericentromeres and their organization can greatly vary between organisms, or even between chromosomes of the same species (Figure 1B). This suggests that pericentromeric functions might not depend on a specific DNA motif recognized by sequence-specific DNA binding machineries. This also suggests that pericentromeric functions could be epigenetically regulated, as is the case for centromeres. Unlike the critical need for telomeres, the importance of pericentromeric regions is unclear in metazoans, and their presence or abundance may not confer any benefit. The genetic ablation of these loci, while technically challenging, would be key to assigning a function for these elements. Nonetheless, these loci must remain under control because in various abnormal situations, like cancer, defective heterochromatic activities can result in chromosomal rearrangements involving pericentromeric regions [3]. It is therefore important to understand how heterochromatin regulates this region.

Historically, pericentromeric heterochromatin has been viewed as an unvarying and static structure, in which only few regulatory processes occur. However, progresses in the analysis of histone modifications, proteomics, and transcriptomics, are changing this view. In fact during development or in disease, distinct protein complexes are recruited to pericentromeric heterochromatin, reflecting unexpected plasticity. Moreover, there is increasing evidence that pericentromeric satellite repeats are expressed in a multitude of organisms, in various biological contexts, and, possibly, in a controlled strand-specific manner. These data suggest that the regulation and the formation of constitutive heterochromatin domains may be more dynamic than anticipated.

Therefore, understanding the biogenesis and the function of heterochromatin at pericentromeric regions is of fundamental interest, and could shed light on the epigenetic regulation of other chromosomal processes. In the first part of this review, we will give an overview on the formation and maintenance of constitutive heterochromatin, with a focus on epigenetic marks, their putative functions, and their responsible enzymes or complexes. The second part of the review will describe and discuss putative functions of pericentromeric transcription and RNA species. Finally, we will discuss research directions that we think should be taken in order to understand the function of this large part of the genome.

### Pathways involved in constitutive heterochromatin formation at pericentromeric regions

**Figure 2:**
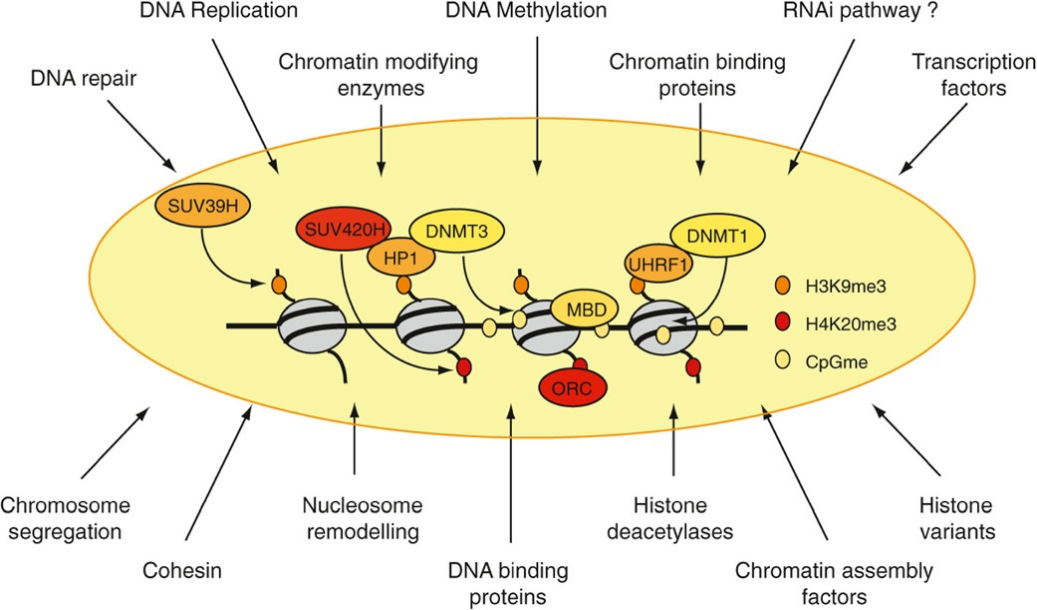
**Schematic representation of constitutive heterochromatin formation in mammals.** SUV39H is the responsible HTMase for H3K9me3 on pericentromeres, a histone mark recognized by HP1 proteins. HP1 proteins interact and recruit SUV420H and DNMTs, leading to H4K20me3 and DNA methylation, respectively. These epigenetic marks function also as docking sites, like H4K20me3 for ORC (origin of replication complex) proteins and CpGme for MBDs (factors with a methyl-binding domain). An alternative for DNMT recruitment might be through UHRF1 that directly interacts with DNMT1 and might read the H3K9me3 mark. In general, proteins involved in various pathways are required for heterochromatin formation and maintenance, and are listed in this panel.

As mentioned, pericentromeric loci do not have strong sequence conservation and do not harbor notable functional genic features (for example, promoter elements) that could trigger heterochromatin formation. At the sequence level, a conserved characteristic is the tandem iteration of DNA motifs. The repetition might be a critical feature for heterochromatin formation and maintenance, as tandemly repetitive transgenes in flies and in plants can be silenced, but the mechanism is unknown. To identify genes whose products enforce heterochromatin, the expression of a reporter localized at pericentromeric heterochromatin was measured in mutagenized backgrounds in *Drosophila* (suppressor of variegation screens). Those early screens, also more recently developed in a mammalian context (*modifiers of murine metastable epialleles* screens), identified factors that enforce heterochromatin [4]. Altogether, with further biochemical studies, about 50 proteins have been found enriched at pericentromeric regions (for review [5]). These proteins include various transcription factors, some histone variants, the linker histone H1, chromatin remodelers, histone modifying enzymes, chromatin binding proteins, DNA methylation enzymes, DNA methyl-binding proteins, and several proteins know to be involved in replication or in the control of the cell cycle [6, 7]. These factors are believed to interact to form a compact structure. However, the respective contribution of each of these activities to heterochromatin formation is poorly understood. Of note, none of these screens uncovered non-nuclear proteins, which might suggest the absence of signal transduction pathways specific to the control of pericentromeric heterochromatin. We list next the major pathways involved in the control of pericentromeric heterochromatin in mammals. Activities controlling pericentromeric heterochromatin are outlined in Figure 2.

### Heterochromatin protein 1 is a major component of constitutive heterochromatin

Heterochromatin protein 1 (HP1) is the first heterochromatin factor identified as a dosage-dependent modifier of position-effect variegation in *Drosophila* [8, 9]. Heterochromatin protein 1 is a small protein that is conserved in eukaryotes except in budding yeast. Not only was HP1 the first heterochromatic factor identified, but it also turned out to be a central component. In fact, through its ability to associate specifically to heterochromatic nucleosomes and to a diverse set of factors [10]. HP1 is believed to propagate the heterochromatic state and to coordinate multiple activities at heterochromatin, including silencing, cohesion, and replication (see next).

### Histone lysine methylation in heterochromatin formation and maintenance

Histone methylation is a conserved and prominent feature of heterochromatic regions. This modification occurs mostly on lysine and arginine residues of histone tails [11]. Lysine methylation can exist in three flavors, mono-, di-, and trimethylation; the methylation index plays important roles in modulating downstream signaling events [11–13]. Depending on the context, this modification can be installed and removed by two antagonizing sets of enzymes, lysine methyltransferases (KMTs), or ‘writers’, and lysine demethylases (KDMs), or ‘erasers’ [14]. As the histone code hypothesis posits [15], these marks can be specifically recognized by protein complexes (or ‘readers’) or can exclude association of unwanted protein machineries. Methylation of H3K9, H3K27, and H4K20 is usually associated with gene silencing and is mostly found at heterochromatic regions. We outline next their biogenesis and putative functions at heterochromatin.

### Biogenesis and function of H3K9me3 at pericentromeric heterochromatin

The monomethylation of H3K9 at pericentromeric heterochromatin relies on the action of two enzymes (Prdm3 and 16), and this reaction might occur prior to histone deposition [16]. Another KMT, SETDB1 has also been shown to be involved in this process [17]. Amongst the other known mammalian H3K9-specific lysine methyl-transferases, the two SUV39H1/2 (also called KMT1A/B) mediate di- and tri-methylation of H3K9 specifically at pericentromeric heterochromatin. This modification has emerged as the hallmark of constitutive heterochromatin in most eukaryotic species, and as described here, this mark lies upstream of other heterochromatin characteristics and therefore controls them. Of note, H3K9me3 is specifically recognized by HP1 [18]. The genes encoding for the responsible proteins were identified early in suppressor of variegation screens (hence their name), although their molecular function was initially not understood. The loss of *Su(var)3-9* (*Suv39h* in mammals) function leads to a specific loss of H3K9me3 at pericentromeric regions but not at other regions marked by H3K9me3, suggesting a specific targeting mechanism. This loss is accompanied by a slight increase in pericentromeric transcription [19]. H3K9me3 levels at pericentromeres vary throughout the cell cycle. Mitosis-specific changes have been shown, with rapid increases when the cell enters mitosis. Maximum levels are reached in metaphase, which decrease once the cell exits mitosis [20]. The significance of these variations is not known.

In mouse somatic cells, the bulk of H3K9me3 co-localizes with DAPI-dense foci (Figure 1C), which stain the array of A/T-rich major satellites that constitute most of the pericentromeric loci. *Suv39h*^*-/-*^ mice are viable, which suggests that the enzyme’s functions may not be critically required for development and differentiation. However, *Suv39h* null mice harbor some lethality in later stages of embryonic development and have altered cell viability. The mutant phenotype includes abnormal chromosomal segregation, disruption of spermatogenesis (hypogonadism and fertility loss) and increased risks of tumorigenesis [21]. Whether aberrant pericentromeric regulation is directly involved in these defects remains unclear.

In *Drosophila*, it was shown that *Su(var)3-9* is also essential for maintaining nucleolar stability and that loss of *Su(var)3-9* causes fragmentation of the nucleolus. This has been attributed to aberrant recombination of repeated DNA sequences, resulting in instability of the rDNA locus. This event can be assessed by measuring the levels of extrachromosomal circular (ECC) DNA [22]. Increased ECC DNA formation due to instability of the rDNA locus has been shown to cause accelerated aging in yeast and *Drosophila* [23, 24]. In mammals, it has been shown that the heterochromatin proteins SUV39H and SIRT1, a histone deacetylase (HDAC) also silence rDNA transcription in response to changes of intracellular energy status [25, 26]. However, ECC DNA has not been documented in *Suv39h*^-/-^ cells in mammals.

How SUV39H proteins are targeted to chromatin in the first place is unclear, but once they are present it is believed that the recruitment is sustained by H3K9me3. In fact, this modification serves as a docking site for SUV39H, which contains a specialized domain called the chromodomain, able to bind to H3K9me3. The H3K9me3 marks serve also to stabilize the recruitment of another major chromodomain protein named HP1 [27]. Because HP1 and SUV39H also interact with each other, this rather simple network forms the basis of a self-assembly mechanism for constitutive heterochromatin maintenance. While in mammals the recruitment of SUV39H and the formation and spreading of heterochromatin at pericentromeres is only poorly understood, this critical question of establishment is better understood in *Schizosaccharomyces pombe*, and involves the RNA interference (RNAi) machinery (see next).

### Biogenesis and function of H4K20me3 at pericentromeric heterochromatin

Another hallmark of pericentromeric heterochromatin is the strong enrichment in the H4K20me2/3 mark. However, the function of this mark remains unclear. H3K9me3 is required for the induction of H4K20me2/3 by SUV420H enzymes (also called KMT5B/C). The interaction of SUV420H and HP1 isoforms (α, β, and γ) explains how the enzyme is targeted to heterochromatin. Although it has been named a suppressor of variegation, the function of the enzyme and the H4K20me3 mark in suppressing transcription is poorly known. In fact, the role played by this enzyme as a genuine suppressor of variegation has been questioned [28]. If not involved in silencing, what could be the function of SUV420H?

During mitosis, pericentromeric heterochromatin is believed to be important for facilitating sister-chromatid cohesion by recruiting cohesin complexes [29, 30]. Several cohesin subunits were shown to interact with SUV420H2 and this interaction is necessary for cohesin binding to heterochromatin [31]. This suggests that SUV420H2 plays essential roles in regulating nuclear architecture and ensuring sister-chromatid cohesion and proper chromosome segregation [32, 33].

Our unpublished data on the function of SUV420H at pericentromeric chromatin of mouse embryonic stem cells suggest that heterochromatin, as defined by H3K9me3 and DNA methylation and their associated machineries, is not overtly perturbed by the absence of SUV420H proteins. The functions of this mark may become critical in other cell types or when cells are challenged by external stimuli.

### Biogenesis and function of other histone lysine methylations at pericentromeric heterochromatin

In addition to H3K9me and H4K20me, there are two other methylation marks, set on H3, that are found enriched at pericentromeric heterochromatin. One is H3K27me1, and it is currently unclear which machinery is involved in setting this mark [19]. It has been shown that the G9A complex, critical to install H3K9me1 and 2 at euchromatic loci, could also induce H3K27me1/2 *in vitro* or *in vivo* [34, 35]. Alternatively, the Polycomb repressive complex 2 (PRC2), usually responsible for installing H3K27me2 and 3 could also induce H3K27me1. Nonetheless, the biological significance of H3K27me1 is currently unknown, although a role in replication in *Tetrahymena* has recently been explored [36]. The H3K64me3 mark is another modification described more recently, but here again both the biogenesis and the function of this mark are unclear [37].

### Histone variants at pericentromeric regions

ATRX, a member of the Swi2/Snf2 family of chromatin-remodeling complexes [38], has been found enriched at pericentromeric heterochromatin [39]. ATRX binding is cell-cycle independent; however, the transcription repressor DAXX (death domain-associated protein) and the histone chaperone SSRP1 (structure-specific recognition protein 1, a subunit of the FACT-complex) are actively recruited to pericentromeres in late S phase, and this recruitment depends on ATRX phosphorylation [40]. As ATRX and DAXX seem to be involved in the deposition of the histone H3.3 variant at repetitive regions [41], it is tempting to speculate that histone composition is modified at pericentromeric regions after replication. The specific role of H3.3 at pericentromerics region is unclear but might be linked to transcription of the locus, which in turn has been correlated with HP1 recruitment [42, 43]. In the same vein, another histone variant H2A.Z has been demonstrated to be critical for HP1 recruitment at pericentromeric loci during development [44, 45].

### DNA methylation

In many organisms, DNA methylation regulates heterochromatin and gene expression. In mammals, this mark mostly occurs in the CpG context, although non-CpG methylation exists, especially in embryonic stem cells. Three catalytically active DNA methyltransferases (DNMTs) have been described. DNMT1 is the maintenance DNMT and is involved in propagating heritable DNA methylation patterns following DNA replication. DNMT3A and DNMT3B are *de novo* DNMTs, which are highly expressed during embryogenesis and are usually deregulated in cancer cells [46]. Their activity is reduced during differentiation. In adult tissues, the expression of DNMT3A is ubiquitous, while DNMT3B is expressed at very low levels [47]. The function of DNA methylation at mammalian pericentromeric heterochromatin is unknown, but perturbing this mark has profound consequences on the epigenetic programming of this locus [48].

H3K9me was originally shown to be a prerequisite for DNA methylation in *Neurospora crassa* [49] and in plants [50]. H3K9me3 and DNA methylation systems also interact at mammalian pericentromeric heterochromatin. *Suv39h* knockout mouse cells reveal an altered DNA methylation profile, specifically at pericentromeric satellite repeat sequences [51]. To explain the crosstalk between the two pathways, a physical and regulatory link between HP1 (recruited by H3K9me3) and DNMT3B has been documented at pericentromeric heterochromatin. Moreover, UHRF1 (also called NP95 in mice) links the two epigenetic methylation tags, as it specifically binds to H3K9me3 and also recruits DNMT1 [52, 53]. Along this line, we observed a reduction in pericentromeric DNMT1 in *Suv39h ko* mouse embryonic stem cells. Conversely, H3K9me3 levels at pericentromeric heterochromatin are not reduced in *Dnmt1* or *Dnmt3a*/*Dnmt3b* single or double knockout cells [51]. We have recently shown, in mouse embryonic stem cells, that the complete removal of DNA methylation significantly affects H3K9me3 levels, disrupts pericentromeric architecture, and leads to a reprogramming of the locus into a Polycomb-regulated region [48].

The role of DNA methylation in preventing pericentromeric transcription is unclear. Transcriptional repressors like MeCP2 are indeed directly recruited by methylated DNA, but no increase in steady-state pericentromeric RNA was observed in *Dnmt*-deficient mouse embryonic stem cells. Conversely, tumor cells, which usually harbor hypo-methylated DNA at pericentromeric loci, show massive transcription of this locus.

### Chromatin-remodeling complexes acting at heterochromatin

ATP-dependent chromatin-remodeling complexes are evolutionarily conserved from yeast to human beings. They alter the chromatin state by inducing nucleosome sliding and dissociating histones from DNA, thereby controlling access to DNA [54]. The nucleosome remodeling and deacetylase (NuRD) complex combines ATP-dependent nucleosome remodeling ATPase (either the CHD4 (Mi2-β) or CHD3 (Mi2-α) subunit) with histone deacetylation activity (HDAC1 and HDAC2), another hallmark of silent chromatin. It is localized with specific segments of pericentromeric heterochromatin, consisting of SatII/III DNA located on human chromosomes 1, 9, and 16 in some cancer cell types [55]. Interestingly, it was found that the expression of several subunits of the NuRD complex is reduced in cells from patients with Hutchinson-Gilford progeria syndrome and in aged cells, which coincided with the loss of heterochromatin markers and increased levels of DNA damage markers γH2AX [56]. This suggested that the NuRD complex prevents DNA damage accumulation, presumably by preserving higher-order structures.

The nucleolar remodeling complex NoRC consists of SNF2H (sucrose nonfermenting-2 homolog), and TIP5 (TTF-I-interacting protein 5), a member of the imitation switch (ISWI)/Snf2h family of remodeling factors [57]. It was shown that NoRC establishes a repressive chromatin environment at heterochromatin and in particular at centromeres [58]. NoRC complex remodels nucleosomes, which promotes heterochromatin formation, and recruitment of HDAC, HMTase, and DNMT activity [59], although how this is achieved is unclear.

The ISWI chromatin-remodeling complex is also required for replication through heterochromatin in mammalian cells. There is evidence that ACF1 (ATP-utilizing chromatin assembly and remodeling factor 1) in complex with SNF2H is required for efficient DNA replication through pericentromeric heterochromatin and may facilitate this process by remodeling the chromatin structure to allow the progression of the replication fork [60].

Human helicase lymphoid specific (HELLS; also called LSH for lymphoid specific helicase and SMARCA6) belongs to a subfamily of SWI/SNF chromatin-remodeling complexes [61], and preferentially localizes to pericentromeric heterochromatin. It assists and maintains histones methylation and acetylation levels at the pericentromeric heterochromatin [62]. Interestingly, while levels of H3K9me3 are maintained in mouse embryonic fibroblasts deleted for *HELLS,* dramatic changes in H3K4me and acetylation levels at the pericentromeric heterochromatin have been reported [62, 63]. This increase in acetylation was dominant in all heterochromatin repetitive elements but there was no change in histone acetylation levels in any gene promoters, suggesting that HELLS plays a specific role in protecting histone acetylation levels only at repetitive elements in the genome [64].

Finally, the SMARCAD1 protein, a protein related to the SWI/SNF family of nucleosome remodelers, has also been shown to be important for heterochromatin replication by deacetylating histones after their deposition by replicative chaperones [65].

### Polycomb proteins

Polycomb proteins form hetero-multimeric complexes that are essential regulators of lineage choices during differentiation and development. In mammals, these complexes also play important roles in cell proliferation, stem cell differentiation, cancer, genomic imprinting, and X chromosome inactivation [66, 67]. PRC2 contains the H3K27 methyltransferase EZH2 (also called KMT6A), as well as EED, SUZ12, RbAp46/48 (RBBP4/7), and JARID2 [68–70]. Canonical PRC1 contains the E3 ubiquitin ligases RING1B (RNF2 in mice) that mediates H2AK119Ub and Polycomb that binds to the H3K27me3 through its chromodomain.

H3K27me3 and H2AK119ub are repressive marks usually associated with transcriptional inhibition at facultative heterochromatin [71] and are not expected to bind to pericentric heterochromatin, where no developmental genes are located. However, Polycomb proteins and activities can be detected at pericentric regions under several specific circumstances. Paternal pericentromeres, which are initially devoid of H3K9me3 marks, transiently recruit PcG proteins after fertilization and until the morula stage [72]. Also mouse cells, in which *Suv39h* genes have been knocked out, show a recruitment of PRC2 activities. Finally, some human cancer cell lines harbor Polycomb nuclear bodies, which are actually constituted of pericentric heterochromatin. It seems that when H3K9me3 activity is impaired (for physiological or pathological reasons), the Polycomb system is recruited to compensate and maintain a heterochromatic environment. The mechanisms underlying such compensation are currently unknown, but the interplay between distinct histone lysine methylation systems reveals a surprising plasticity in propagating methylation patterns in chromatin and offers important insights into fundamental biological processes.

### Hypoacetylated histones at heterochromatin

In the budding yeast *Saccharomyces cerevisiae*, H3K9 or 27 methylation does not exist, and therefore it is often viewed as a species devoid of any heterochromatin. Silent chromatin necessitates the histone deacetylase Sir2 [73–76]. In mammals, seven genes are homologous to Sir2 (SIRTUINS: SIRT1-7). They are localized in the nucleus, cytoplasm, or mitochondria, are specific for different substrates, and therefore have a broad spectrum of functions. SIRT1 in mammals, the Sir2 ortholog, is involved in the regulation of chromatin metabolism, apoptosis, and aging [77, 78]. Cells deficient for SIRT1 encounter an overall increase of H4K16ac and H3K9ac, as well as a loss of epigenetic marks at constitutive heterochromatin, such as H3K9me3 and H4K20me1 [79]. This suggests that SIRT1 is involved in the formation of constitutive heterochromatin in a direct or an indirect manner.

### Transcription factors at heterochromatin

As mentioned earlier, the DNA sequence of pericentromeric loci is not well conserved. Nonetheless, these regions potentially represent a large area onto which DNA binding factors can associate. This is particularly true for transcription factors, which are often sequence-specific DNA binding proteins. In mice, several transcription factors have been shown to bind to pericentromeric regions [80]. Interestingly, our purification of human pericentromeric heterochromatin from diverse cell lines and tissues has led to the identification of very different transcription factors harboring zinc finger domains (unpublished observations). The contribution of transcription factors to heterochromatin formation is unclear, but the absence of promoter elements in these regions has led to the hypothesis that the iterated binding of these factors outside a genic context is in fact critical for heterochromatin formation and silencing [80]. As, depending on the context, transcription factors can recruit co-repressor complexes (for example, NuRD), a model for heterochromatin formation based on transcription factor binding is emerging. Recently, we have shown that the epigenetic status of mouse pericentromeric heterochromatin in fact relies on the ability of DNA binding proteins to associate. The BEND3 factor, which is a methylation-sensitive DNA binding protein, allows the unmethylated locus to be reprogrammed into a PcG regulated locus in mouse embryonic stem cells, possibly via recruiting the NuRD complex [48]. Thus, transcription factor binding certainly plays a critical role that will require more extensive research efforts.

### Heterochromatin formation in fission yeast versus mammals

**Figure 3:**
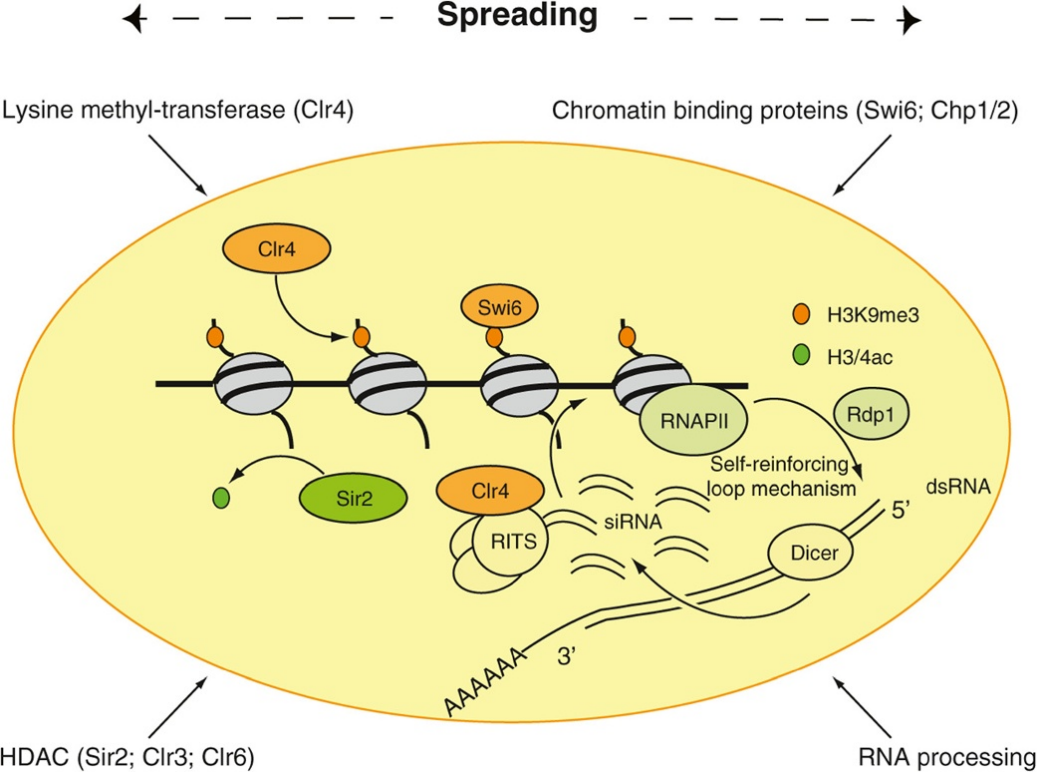
**Schematic representation of constitutive heterochromatin formation in fission yeast.** Sir2 is responsible for deacetylation of histone tails. Clr4 methylates histone H3 on lysine 9 (H3K9me3), which is an anchor for the HP1 homolog Swi6. RNA Polymerase II (RNAPII) transcribes pericentromeric noncoding repeats in single strand RNA. Rdp1, a component of the RNA-dependent RNA polymerase, can generate a double-strand RNA, which is digested by Dicer to produce short interfering RNAs (siRNAs). The siRNAs associate with the RITS (RNA-induced initiation of transcriptional silencing) complex, which is responsible for further recruitment of Clr4, and therefore stimulates further H3K9me3 and maintains local heterochromatin.

During the last decade, great progress has been made in understanding how heterochromatin forms in *S. pombe*. The basic mechanism implicates components involved in chromatin modifications, like histone deacetylases Clr3 (HDAC1), Clr6 (RPD3), and, especially, Sir2 (which deacetylates H3K9 and H4K16), which is followed by H3K9me by Clr4 protein (a homolog of mammalian proteins SUV39H) (reviewed in [81]) (Figure 3). H3K9me acts as a docking site recognized by the chromodomain of Swi6, Chp1, and Chp2 proteins (HP1 homologs). The spreading of heterochromatin also occurs via the Sir2 protein, which deacetylates new histones to allow the recruitment of Clr4 and Swi6 proteins [82]. Finally, the RNAi proteins Argonaute and Dicer are critically required for heterochromatin formation in *S. pombe* [83], and for the initial targeting of Clr4.

Since most involved proteins have orthologs in metazoans, the hypothesis that heterochromatin forms using similar mechanisms in mammals is very popular. Chromatin-modifying activities, including DNMT, HMTase, and HDAC chromatin remodelers, amongst others including NuRD, ATRX, and NoRC, and transcription factors and co-repressors, like Gfi1b, Sall1, Trim28, and Pax3-9, were reported to be involved in heterochromatin formation in mammals (Figure 2). However, particularly in mammals, there is very little evidence that RNAi components are involved in the establishment of heterochromatin. It may be that the contributions of these factors are only critical at developmental stages, where heterochromatin is established (for example, in the zygote). In fact, double-stranded RNA was suggested to play a role in HP1 recruitment at this stage [43].

### Transcription of pericentromeric heterochromatin

**Figure 4:**
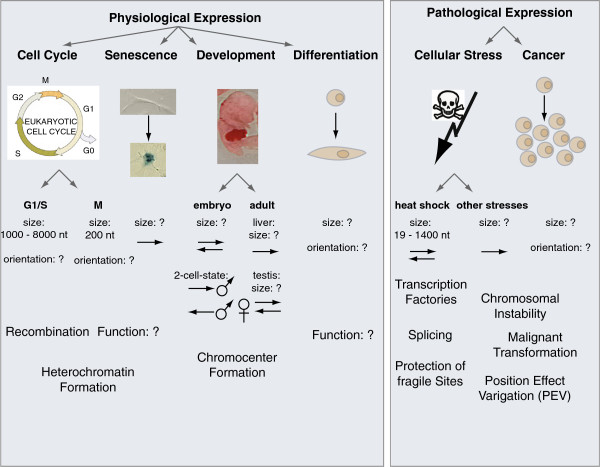
**Different biological contexts of pericentromeric satellite expression.** Physiological expression of pericentromeric satellite repeats has been reported during cell cycle, aging cellular senescence, differentiation and development. Expression levels are detectible but low. Pathological expression has been reported upon cellular stress and in cancer, and expression levels are often aberrantly overexpressed. The size and orientation of the transcripts are indicated when known. In addition, putative functions of the noncoding transcripts in different biological contexts are mentioned and discussed in the text.

The aforementioned proteins, which act at heterochromatin, have often been defined by their ability to drive transcriptional gene silencing. Very early (1969) RNA-DNA hybridization experiments have suggested that pericentromeric DNA was transcriptionally silent in differentiated mouse tissues [84]. However, in the same period, the first indications of a possible satellite DNA transcription in mouse tumor cells were presented [85]. Today, with the increased sensitivity of molecular techniques, transcription of pericentromeric satellite repeats has been confirmed in a multitude of organisms and in various contexts, including proliferation, development, differentiation, senescence, stress response, and transformation. This could either be the consequence of leaky heterochromatin, or it might reflect specific, and perhaps, conserved, functions of the transcription process or of the resulting transcripts (Figure 4) (reviewed in [86–88]).

In general, pericentromeric satellite transcripts vary in length and are considered as nonprotein coding. Compared with the amount of satellite repeats, from which they are generated (from 2% to >10% of the genome), satellite RNAs are usually not very abundant in somatic cells, suggesting that transcription is a relatively rare event, or that RNAs are highly unstable. They are produced by RNA Polymerase II (RNAPII) and can exist in sense (in mice: T-rich; in human beings: GGAAT) and antisense (in mice: A-rich; in human beings: ATTCC) orientations (Figure 4) [89, 90]. The fact that sense and antisense transcripts are not necessarily present in equal quantities also suggests that transcription might underlie a regulated process that is controlled by specific regulatory DNA elements rather than an unspecific side-product of decondensed pericentromeres. Until now, a few factors involved in transcriptional activation have been identified in human beings and under specific stress conditions. These include Heat-Shock Factor 1 (HSF1) (upon heat stress) and tonicity Enhancer-Binding Protein (TonEBP) (upon osmotic stress) [91, 92]. Several studies have also correlated transcriptional activation of pericentromeric satellites with decondensation of heterochromatin that goes along with an increase in activating histone marks [90]. In mouse cells, increases in pericentromeric transcription have been correlated with a failure to incorporate the replication-independent H3.3 histone variant at pericentromeres [42]. Owing to the repetitive nature of pericentromeres, quantitative studies of DNA methylation levels as well as histone modification profiles are difficult, and only a little information concerning possible changes in the epigenetic status is available. Since most biological readouts, in which pericentromere transcription has been observed, underlie changes in epigenetic mechanisms, we suggest that such mechanisms could control pericentromere transcription. Interestingly however, it was shown that pericentromeric transcription could occur in the presence of high levels of repressive marks (H3K9me3 and H4K20me3) [93, 94].

Mechanistic insights into the role of pericentromeric transcription are better described in *S. Pombe*. In *S. Pombe*, double-stranded RNA formed from long single-stranded pericentromeric transcripts can be cleaved by Dicer to form short interfering RNAs (siRNAs). The siRNAs associate with the RNA-induced initiation of transcriptional silencing (RITS) complex, which is responsible for the recruitment of the histone methyltransferase Clr4 that methylates H3K9 and therefore maintains local heterochromatin (reviewed in [95, 96]). In general, transcripts exist in an antisense orientation; however, during the S phase, an increased presence of sense transcripts has been observed [97]. Similar pathways to establish heterochromatin at pericentromeric heterochromatin have also been identified in plants [96, 98].

The role of RNAi in heterochromatin formation is less obvious in mammals and remains a controversial issue. Two independent studies suggested Dicer dependent pericentromeric RNA processing in mammals, which, when impaired, leads to the accumulation of satellite transcripts of various sizes and severe differentiation defects and cell death [99, 100]. However, small double-stranded RNAs that originate from pericentromeres could not be detected in many other mammalian cell systems. It could be that RNAi is only involved in the establishment and not the maintenance of heterochromatin and that this system was only assessed in tissues or cell lines, where heterochromatin is maintained only. Alternatively, mammalian organisms have developed other mechanisms to ensure heterochromatin formation. Another (less likely) possibility, is that RNAi could not be detected because of the very low levels of small double-stranded RNAs. Irrespective of the controversial opinions on the involvement of RNAi in mammals, pericentromeric transcripts could indeed be shown to be involved in heterochromatin formation and maintenance. For instance, it was shown that WDHD1 (WD repeat and HMG-box DNA binding protein 1) interacts with major satellite transcripts in mice. WDHD1 plays a role in RNAPII transcription and RNA processing. Importantly, upon depletion of WDHD1, transcription of major satellites was increased while condensation of heterochromatin at this locus was decreased, resulting in proliferation defects [101]. One of the most convincing pieces of evidence for a role of RNA in heterochromatin formation comes from a work in the mouse early embryo: injections of satellite double-stranded RNA are able to target HP1β to pericentromeric regions, suggesting the RNA targets HP1 to heterochromatin in a sequence-specific manner; however, the connection with the RNAi machinery was not explored [43]. As with most other noncoding transcripts, detailed molecular insights into the mechanism of action at chromatin are lacking. Moreover, in the case of pericentromeric transcripts, clear biological roles have not yet been identified and transcription could be the consequence of imperfect heterochromatin silencing, with no particular function. Several other interesting hypotheses of potential functions have been proposed, and are described according to their biological context.

### Transcription in proliferating cells during the cell cycle

Lu and Gilbert found a physiological transient transcription of pericentromeric heterochromatin in primary mouse embryonic fibroblasts, which was proliferation and cell-cycle dependent [102]. A first wave of major satellite transcription was observed in the late G1-phase, with a peak in the early S phase. Transcription was regulated by RNAPII; the resulting transcripts were highly heterogeneous and varied in length from 1000 to more than 8000 nucleotides. As transcription occurred just before replication of pericentromeres in the mid- or late S phase, and transcripts accumulated at the place of pericentromere replication, it was suggested that pericentromeric transcription could be involved in preparing heterochromatin for reassembly at the replication fork.

Interestingly, a second wave of pericentromeric transcription was observed in the M-phase, which is rather intriguing, as RNAPII transcription is generally shut down during mitosis. The resulting transcripts were smaller (200 nucleotides), and coincided with chromatin clearing from transcription factors and other associated proteins. It was suggested that this specific population could indeed be involved in heterochromatin formation or maintenance [103–105].

It was shown that HP1 is evicted from heterochromatin during the M-phase by a mechanism called methyl-phospho-switch [103, 105]. In addition to H3K9me3, which is recognized by the HP1 through its chromodomain, HP1 is also tethered to heterochromatin by an RNA component [104]. Thus, the short pericentromeric RNAs, transcribed during the M-phase, could play a role in recruiting HP1 to heterochromatin after mitosis. The significance of such cell-cycle dependent differences in transcription remains unknown. It could be argued that these dynamic changes are merely a consequence of variations in chromatin organization and compaction during mitosis, but the idea that complex phenomena like these have a biological purpose is tantalizing.

### Transcription during development and differentiation

Spatially and temporally regulated activation of pericentromeric transcription was observed throughout mouse development [89]. For instance, an accumulation of antisense satellite transcripts was found in the central nervous system of embryos 11.5 dpc (days post coitum), which from 12.5 dpc until 15.5 dpc was replaced by an accumulation of sense transcripts. In adult mice, expression of pericentromeric transcripts was only found in liver and testis, but not in any other tissue, including the brain [89]. Interestingly, liver and testes are highly proliferative tissues, which suggests once more a link between cell-cycle progression and pericentromeric transcription. In the liver, transcripts exclusively existed in the sense orientation, while in the testis, transcripts were observed in the antisense orientation in immature germ cells and in the sense orientation in mature germ cells, again suggesting a regulated process during differentiation.

Probst and co-workers [106] performed a detailed study at the two-cell stage of the mouse preimplantation embryo and found that pericentromeric transcripts are important at this stage. Chromocenters are nuclear structures formed by the aggregation of heterochromatin from multiple chromosomes. Interestingly, at the two-cell stage, transient transcription of pericentromere satellite repeats seemed to be spatially and temporally regulated and coincided with the reorganization of pericentromeric satellite DNA into chromocenters. At the beginning of the two-cell stage, pericentromeres were only transcribed form the paternal chromosome in the sense orientation. Once chromocenters had formed at the end of the two-cell stage, there was a burst of pericentromere transcription from the antisense strand and from both the maternal and paternal chromosomes. While sense transcripts were localized in the nucleus and the cytoplasm, antisense transcripts were exclusively found within the nucleus. After the second mitotic division, major satellite transcription was rapidly shut down again [106]. Pericentromeric transcripts have also been observed in various cell differentiation models. Those include terminal muscle cell differentiation and mouse embryonic stem cells undergoing retinoic-acid-induced differentiation [93, 94]. Resulting major satellite transcripts were also located at chromocenters and could account for their formation [87, 107]. The dynamics of strand-specific transcription, and the differential processing and localization of such transcripts constitute another example, building a stronger case for a biological function of pericentromeric transcription.

Regulated pericentromeric transcription has also been reported during epithelial mesenchymal transition, the conversion of epithelial cells into mesenchymal cells. This process is critical during embryonic development and also during cancer progression. Two important players are the transcription factor Snail1 and the H3K4me3 specific deaminase LoxL2 [108]. At the onset of epithelial mesenchymal transition, SNAIL1 is rapidly upregulated and recruits LOXL2 to oxidize H3 at pericentromeres, leading to a transient downregulation of major satellite transcription. Interestingly, this coincides with a transient release of HP1α [109]. Once epithelial mesenchymal transition has completed, HP1α binding and major satellite transcription are re-established in mesenchymal cells.

### Transcription in cellular senescence

Transcriptional activation of pericentromeres has also been observed in replicative senescence and aging [110], where it becomes clearer that profound epigenetic changes occur throughout the genome. Upon extensive passaging of primary human fibroblasts, cells entered replicative senescence, which correlated with expression of pericentromeric transcripts that were polyadenylated and in a sense orientation. In addition, constitutive heterochromatin at pericentromeres was decondensed and revealed lower DNA methylation levels. Here, the transcripts might not serve a specific function and could result from senescent heterochromatin.

### Transcription upon cellular stress

Pericentromeric transcription has been reported upon various cellular stresses, including heat shock, exposure to heavy metals, hazardous chemicals, and ultraviolet light, as well as hyperosmotic and oxidative conditions, and is up to now one of the best studied contexts of pericentromere transcription [87, 92, 111, 112]. Interestingly, expression levels vary according to the nature of the cellular stress, with heat shock being the strongest inducer. In addition, only upon heat shock, transcripts in both orientations have been observed, however, with sense transcripts being more prominent. All other cellular stresses induced transcription in the sense orientation only [92].

Upon heat shock of human cells, nuclear stress bodies (nSBs), distinct nuclear structures, are formed on pericentromeric regions. In particular, the large pericentromeric region of chromosome 9 (9q12) was shown to be tethered within nSBs [113, 114]. Interestingly, the epigenetic status of pericentromeres within nSBs was changed and bore characteristics of euchromatin, including hyper-acetylation of histones. In addition, HSF1 and RNAPII were both recruited to nSBs, which correlated with transcription of specific pericentromeric satellite repeats (a block of SatIII repeats located at pericentromeric regions of chromosome 9). The resulting polyadenylated transcripts were very large, in the sense orientation, and remained associated with nSBs, the site of their transcription [91, 112]. A detailed study to characterize structurally and functionally SatIII transcripts in heat-shock cells revealed that each satellite transcript has a unique structure [90]. They are composed of classical SatIII repeats and are of varying length (19 to 1400 nucleotides). Knock-down of these transcripts by antisense oligonucleotides and by RNAi affected the recruitment of RNA processing factors to nSBs, suggesting a role of SatIII transcripts in the self-organization of these nuclear bodies. In fact, recruitment of the splicing factor SF2/ASF was dependent on SatIII transcription [115, 116].

Recently, DAXX, a death domain-associated protein, was shown to play a role in SatIII transcriptional activation upon heat shock. In addition to its main role in apoptosis, Daxx was shown to be a chaperone of the histone variant H3.3 [42]. Upon heat shock, Daxx switched location from PML nuclear bodies to SatIII pericentromeric repeats. When Daxx was depleted from heat-shock cells, the expression of SatIII was less pronounced and correlated with reduced incorporation of H3.3 at pericentromeres [117].

While transcriptional activation of SatIII repeats upon heat shock was shown to underlie the action of HSF1, upon other cellular stresses, different transcription factors could be involved. Accordingly, in cells exposed to hyperosmotic stress, a moderate inducer of SatIII transcription, TONEBP was found bound to pericentromeres. In *TONEBP* loss-of-function studies it was shown that this factor was indeed essential for SatIII expression [92]. Interestingly, hyperosmotic stress, as well as other cellular stresses, also led to the formation of nSBs. The number and size of nSBs, however, was reduced and correlated with reduced SatIII expression levels, compared with heat shock.

A detailed expression study by Eymery and co-workers [1] compared satellite transcription of centromeres and pericentromeres during cellular stress as well as in ‘normal’ and cancer cells and confirmed a strong upregulation of pericentromeric transcripts upon heat shock.

Once again, the function of this specific transcription, or of the RNA, in stress response remains unclear. Nonetheless, some hypothetical suggestions have been made. First, these transcripts could be processed in an RNAi-dependent or -independent pathway and therefore be implicated in heterochromatin re-formation, as has been shown in fission yeast. Second, similar to X chromosome inactivation by the long ncRNA Xist, the long noncoding pericentromeric transcripts might be involved in the establishment or maintenance of a specific chromatin state (yet to be defined). Third, the SatIII transcripts could serve to protect a fragile region of the genome from stress-induced damage, although how this could work remains unclear. Intriguingly, it has been shown that the 9q12 region, which hosts the bulk of SatIII, is often rearranged in certain pathologies, including cancer [91, 118]. Fourth, pericentromeric transcripts could play a role in splicing regulation during stress response by sequestration of splicing factors (reviewed in [119, 120]). In fact, direct interactions of splicing factors with pericentromeric RNA have been shown [113, 115, 116]. However, whether such a phenomenon indeed affects the splicing of cellular genes remains to be explored. Finally, a position-effect mechanism has been proposed, suggesting that activation of pericentromeric satellite repeats could counteract the repressive nature of heterochromatin and activate genes located nearby in *cis* or in *trans*.

In addition to pericentromeric transcription, centromeric transcription has also been widely observed upon cellular stress (reviewed in [1, 86, 88]). For instance, mouse cells exposed to chemical stress revealed increased centromeric minor satellite expression, which correlated with decondensed centromeres. As a consequence, centromere function was impaired and mitotic defects, including multiple spindle attachments and aneuploidy, were observed [121].

### Transcription in cancer and diseases

Under physiological conditions, pericentromeric satellite repeat expression has been observed in proliferating cells and during development. However, in some pathological incidences, misregulation of pericentromeric satellites has been reported, together with decondensation and demethylation of pericentromeric DNA. For instance, aberrant overexpression of pericentromeric satellite repeats has been reported in several epithelial cancers [87, 122, 123]. In addition, decondensation of pericentromeric heterochromatin and transcriptional activation has also been observed in several genetic disorders [3, 124].

The first evidence of the existence of satellite transcripts in cancer originates from findings in Wilms neuroblastoma tumors and epidermal carcinoma cells [110, 125]. Pericentromeric satellite expression was also upregulated in lung cancer, in comparison with healthy lung samples.

Numerous diseases and cancers result, to a great extent, from deregulated epigenetic mechanisms, which could also directly affect the compaction status of pericentromeres and their expression potential. For instance, the lysine-specific demethylase 2A (KDM2A) is a tumor suppressor gene, which is downregulated in prostate cancer [126]. KDM2A is a specific H3K36 demethylase and was also shown to target pericentromeres, thereby ensuring a compact and condensed chromatin structure. Interestingly, when KDM2A was depleted in mouse and human cells, HP1 binding was lost from pericentromeres, which correlated with transcriptional activation of these elements. Consequently, cells encountered segregation defects and an overall genomic instability.

Another study recently linked the *BRCA1* tumor suppressor gene to a role in the repression of pericentromeric expression [122]. Mutations in *BRCA1*, which has an E3 ligase activity in its RING finger domain and can ubiquitinate lysine 119 in histone H2A (H2AK119ub), are one of the main causes of breast or ovarian cancer. *BRCA1* knockout mice revealed a strong increase in major satellite transcripts that correlated with a loss of H2AK119ub at pericentromeres. As a consequence, mitotic defects and increased DNA double-strand breaks have been observed. Ectopic overexpression of satellite RNA in normal cells phenocopied *BRCA1* knockout cells and resulted in genomic instability. Thus, Zhu and co-workers [122] provide evidence that the noncoding pericentromeric transcripts could be a driving force for malignant transformation.

DNA methylation is one of the main epigenetic marks of constitutive heterochromatin at pericentromeres and is a crucial player in transcriptional repression in mammals. Along this line, Sugimura and co-workers [127] observed satellite transcription in mouse embryonic fibroblasts upon 5-aza-dC treatment, a potent inhibitor of DNA methyltransferases. In addition to a strong decrease in DNA methylation levels, transcriptional activation correlated with an increase of active histone marks, like H3K4me3 and acetylation of histone H4 (H4ac), as well as incorporation of the H3.3 histone variant.

Importantly, DNA methylation is impaired in neoplasia, which is characterized by a global DNA demethylation as well as localized hypomethylation of oncogenes and hypermethylation of tumor suppressor genes (reviewed in [128]). Recently, aberrant satellite overexpression in mouse and human epithelial cancers, including cancer of the pancreas, lung, kidney, colon, and prostate, was also linked to deregulated DNA methylation [123]. The use of sophisticated expression methods to analyze the transcriptome of primary tumors uncovered aberrant overexpression of satellite transcripts. In mouse pancreatic ductal adenocarcinoma (PDAC) samples, 47% of all transcripts were mapped to major satellites, while in healthy reference tissues only 0.02 to 0.4% of all transcripts originated from those repeats. Interestingly, PDAC cells revealed only minimal expression of major satellites when cultured *ex vivo*. However, high levels of major satellite transcription, comparable to those observed in tumors, could be triggered by 5-aza-dC, suggesting that transcriptional regulation is dependent on DNA methylation levels, which might be re-established together with other silencing mechanisms *ex vivo*. Moreover, human SatII repeats were 21-fold overexpressed in human PDAC patient samples in comparison with ‘healthy’ tissue samples. Ting and co-workers [123] also identified satellite-correlated genes and revealed that several mRNA encoding genes (involved in neuronal cell fate and stem cell pathways) that mainly contained LINE1 transposable elements were highly expressed. LINE1 insertion upstream of transcriptional start sites of genes can be implicated in their regulation. Indeed, upregulation of several satellite-correlated genes correlated well with proximity of LINE1 insertions. Interestingly, healthy testis tissue showed high expression levels of pericentromeres, while in cancers the expression was silenced [87]. A high expression level of pericentromeric RNA was also observed in adult mice testes [89], suggesting that perturbing the epigenetic state of pericentromeric heterochromatin one way or the other, could lead to cellular transformation.

In addition to cancer, misregulation of DNA methylation at pericentromeric regions could also be causative of immunodeficiency, centromere instability, and facial anomalies syndrome (ICF) [129], in which a majority of patients harbor mutations in one of the three main DNA methyltransferases, DNMT3B. In ICF cells, a severe DNA hypomethylation of SatII repeats in chromosomes 1 and 16, and SatIII repeats in chromosome 9 has been reported [129]. Even though SatII transcripts had been observed in some ICF lymphocytes, the expression levels were low and were not increased, suggesting that hypomethylation is not sufficient for transcriptional activation of these elements [125].

It was proposed that hypomethylation of SatII and SatIII repeats might provoke the deregulation of gene expression in *trans*, by altered sequestration of transcription factors, changes in nuclear architecture, or expression of noncoding satellite transcripts [3].

An aberrant overexpression of pericentromeric transcripts was also observed in the Hutchinson-Gilford progeria syndrome, a premature aging syndrome with a dramatic accelerated aging phenotype beginning in childhood [124].

Hutchinson-Gilford progeria syndrome arises from mutations in the *LaminA* gene (reviewed in [130]). Lamins are structural components of the nuclear lamina and are implicated in the structural integrity of the nucleus. In addition to premature aging phenotypes and changes in nuclear shape and architecture, one characteristic of Hutchinson-Gilford progeria syndrome cells is the constitutive expression of pericentromeric satellite sequences from chromosome 9. The expression of SatIII repeats was shown to correlate with a loss of constitutive heterochromatin mark H3K9me3, and of HP1 binding [124]. A direct link between defective lamina and heterochromatin is still to be demonstrated, but lamins are crucial for pericentromeric heterochromatin organization [131], and this interaction requires a functional heterochromatin [48, 132].

## Conclusion

With the central question of how heterochromatin is established in the first place in mammals, another remaining issue is how transcription can happen inside constitutive heterochromatin, a highly condensed conformation that was believed to be transcriptionally inert. Interestingly, several transcription factors were found to bind into heterochromatic repeat sequences across diverse species. As mentioned already, upon cellular stress, the transcription factors HSF1 and tonEBP were reported to bind to SatIII repeats, as well as the splicing factors SF2/ASF [90, 111–116]. Additionally, factors like GFI1B [133], TRIM28 [134], and SALL1 [135] were shown to bind to the mouse pericentromere region. It is plausible that those transcription factors interplay with RNA polymerase for the expression of pericentromeric satellite repeats within constitutive heterochromatin. It is also unclear how oriented transcription is regulated in the context of promiscuous transcription factor binding. Moreover, the putative function of the transcription process or their resulting transcripts remains elusive. The interactome of satellite transcripts should be investigated, although such approaches may not be insightful: for instance, the identification of factors interacting with noncoding RNA produced at telomeres did not reveal specific functions [136]. Interestingly, pericentromeric transcription has been reported under various conditions (differentiation, cancer, early development, or stress). Thus, the transcriptional regulation can be achieved by distinct pathways for different purposes.

It has been suggested that pericentromeric satellite overexpression could be a driving force in malignant transformation. We speculate that in a large number of diseases, including cancer, the aberrant upregulation of pericentromere transcription correlates with reduced DNA methylation levels at these loci. In addition, decondensation of these loci could also favor DNA breaks and genomic rearrangements, genomic events often observed in cancer.

However, physiological pericentromeric transcription in yeast and even in higher mammals was suggested to be involved in heterochromatin formation and maintenance, therefore ensuring genomic stability. This suggests that these loci stay condensed and in an overall closed chromatin state. If the only function of heterochromatin at pericentromeres is to act as a boundary for centromeres, the oncogenic deregulation of heterochromatin may explain why there is an observed expansion of CENP-A in cancer cells [137].

However, we hypothesize that also the resulting transcripts could be directly involved in the manifestation of the disease, as suggested earlier. Finally, few genes lie within constitutive heterochromatin at pericentromeres, like TPTE, POTE, BAGE, and their aberrant expression could also account for transformation. Additional experiments will be required to shine more light on this aspect.

## Abbreviations

ATRX: α-thalassemia/mental retardation syndrome X-linked protein
BRCA1: breast cancer 1
DAXX: death domain-associated protein
DNMT: DNA (cytosine-5)-methyltransferase
dpc: days post coitum
ECC: extrachromosomal circular
H3K9me3: trimethylated histone 3 at lysine 9
H3K27me: methylated histone 3 at lysine 27
H4K20me3: trimethylated histone 4 at lysine 20
HDAC: histone deacetylase
HELLS: human helicase lymphoid specific
HP1: heterochromatin protein 1
ICF: immunodeficiency, centromere instability, and facial anomalies syndrome
ISWI: imitation switch
KDM: lysine demethylase
KMT: lysine methyltransferase
LSH: lymphoid specific helicase
ncRNA: noncoding RNA
NoRC: nucleolar remodeling complex
nSBs: nuclear stress bodies
NuRD: nucleosome remodeling and histone deacetylase
ORC: origin of replication complex
PDAC: pancreatic ductal adenocarcinoma
PRC2: Polycomb group repressive complex 2
PRC1: Polycomb group repressive complex 1
RITS: RNA-induced initiation of transcriptional silencing
RNAi: RNA interference
RNAPII: RNA Polymerase II
SFN2h: sucrose nonfermenting-2 homolog
siRNA: short interfering RNA
SSRP1: structure-specific recognition protein
SUV39H: suppressor of variegation 3-9 homolog
WDHD1: WD repeat and HMG-box DNA binding protein 1.
